# Massive upper gastrointestinal bleeding – complication of pancreatic pseudocyst

**Published:** 2014-06-25

**Authors:** CI Mavrodin, G Pariza, V Iordache, CS Pop

**Affiliations:** * 3rd Department, Emergency General Surgery, Emergency University Hospital Bucharest; “Carol Davila” University of Medicine and Pharmacy, Bucharest; ** 3rd Department of Internal Medicine, Emergency University Hospital Bucharest; “Carol Davila” University of Medicine and Pharmacy, Bucharest

**Keywords:** pancreatic pseudocyst, upper gastrointestinal bleeding, cystogastrostomy, pancreatitis

## Abstract

Abstract

Massive bleeding is an unusual complication of pancreatitis. Most patients have chronic pancreatic disorders associated with pancreatic pseudocyst. We present the case of a patient, aged 49 years, known with alcohol-induced chronic pancreatitis, corporeal-caudal pancreatic pseudocyst expanded in the omental bursa, admitted to the emergency room because of hematemesis and melena, the endoscopy revealing, as a source, the erosion through the posterior gastric wall by the pseudocyst. The gastrostomy and haemostasis in situ of the source and the pseudocyst-gastric anastomosis was the solution adopted, with favourable long-term evolution.

## Introduction

Intestinal bleeding is a rare complication of pancreatitis but it carries a mortality risk of above 50%. We often discuss about life-threatening massive bleeding, but there are also some insidious forms that associate anaemia [**1-3**]. 

 Bleeding may exteriorize either within the lumen or in the intraperitoneal, retroperitoneal cavity, or simultaneously in the intra- and retroperitoneal space.

 Bleeding complications associated with pancreatitis or pancreatic lesions have as a source either direct pancreatic lesions (pseudoaneurysm or necrosis followed by the rupture of an arterial wall, gastric or oesophageal varices due to splenic vein thrombosis, intracystic bleeding, splenic rupture) or may be caused by coexisting pathologies (peptic ulcer, gastritis, tendinitis, oesophageal varices due to FT, Mallory-Weiss syndrome).

 Erosion caused by the pancreatic inflammatory process or pseudocyst development in an adjacent vessel may lead to the development of a pseudoaneurysm. Its rupture in the gastrointestinal tract can target the pancreatic duct, bile duct, stomach, duodenum or colon and can manifest as haemobilia, upper gastrointestinal bleeding, lower gastrointestinal bleeding [**1,3,,4**]. 

 We present the case of a patient, aged 49 years, with alcohol-induced chronic pancreatitis who presented to our emergency service because of hematemesis, melena and low blood pressure.

**Fig. 1:**
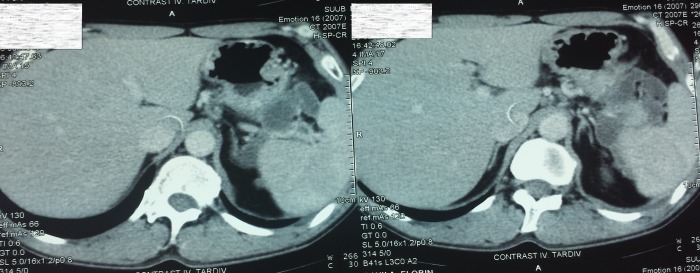
Tomographic aspect of retrogastric pancreatic pseudocyst. Posterior gastric wall thickening, marking the anterior pseudocyst cavity

 On presentation: Hb 8g/dl, BP 100/60, VR 100, hyperamylasemia - 237 U / l. The upper GI endoscopy performed in the emergency revealed the presence of an ulcer in the posterior gastric wall with a diameter of about 1.5 cm, with an adherent clot. Abdominal ultrasound scan and abdominal CT revealed a pancreatic tail pseudocyst in the retrogastric space, with a diameter of 6 cm (**Fig. 1**).

 Under conservative treatment (fluid and electrolyte balance, administration of PRBCs, FFP, antisecretory drugs), the patient repeated the bleeding episode after 5 days, situation that resulted in an altered general condition of the patient with hemodynamic instability (BP 70/40, VR 130/min, Hb 6 g / dl) that required emergency laparotomy.

 Intraoperatively, the presence of a pancreatic tail pseudocyst with a diameter of 6 cm was noticed. It eroded through the posterior gastric wall, and the gastrostomy revealed as a source of bleeding an ulceration corresponding to the erosion area of the pseudocyst in the posterior gastric side, presenting active bleeding. The “in situ” hemostasis was performed, thereby controlling the bleeding source, and the cyst-gastrostomy was performed in the posterior gastric wall by a transgastric approach. Due to the recurrence of the anaemia syndrome accompanied by melena stools, the 10th day after the surgery, the abdominal CT was performed, highlighting the persistency of the pseudocyst. The decision to perform another surgery was made, which revealed bleeding in the cystogastric anastomosis and the presence of blood clots in the pseudocyst. This situation required the removal of the anastomosis, gastrorrhaphy followed by external drainage of pseudocyst cavity.

 Postoperatively, the patient developed low-flow external pancreatic fistula (150 ml / day) that was sorted out conservatively (total parenteral nutrition and Sandostatin), the patient being discharged 24 days following the last operation. The tomography investigation, 12 months postoperatively, did not reveal the presence of pancreatic cystic masses. Clinically, the patient had an uncomplicated incision hernia.

 Discussions

## Discussions

Incidence. Bleeding complications occur in 10-15% of the patients with chronic pancreatitis and in 6-30% of those who develop pancreatic pseudocyst [**5-9**].

 Pancreatic and peripancreatic structures can be destroyed by inflammatory processes and enzyme self-digestion due to pancreatitis and because of pseudocyst adhesion to the pancreas. Furthermore, the arteries and veins in the pseudocyst and spleen wall may be involved in this process. Artery pseudoaneurysms, venous thrombosis and splenic rupture may thus appear [**10-13**]. Pseudoaneurysm can rupture in the intra- retro-peritoneal space and in the gastrointestinal tract. This complication can occur directly or more often as a result of prior bleeding in the pseudocyst cavity, followed by a rupture in the digestive tract. The splenic artery is most commonly affected, followed by the gastro-duodenal, pancreaticoduodenal, pancreatic, gastric and liver artery.

 Angiography can show the presence of a pseudoaneurysm in 10-20% of the patients with chronic pancreatitis without bleeding and in 10-30% of those with a pseudocyst. Most pseudoaneurysms association develop in close proximity to pseudocysts [**6-7**].

 Presentation

 The average age at which haemorrhage associated with pancreatic pseudocyst occurs is 40 years, the clinical picture varies depending on the location and severity of the bleeding, thus presenting in different forms, from anaemia to hypovolemic shock [**3-5**].

 Massive bleeding has been reported in 2-10% of the patients with pancreatitis and occurs as upper or lower gastrointestinal bleeding [**1,6,8,10**]. The rapid development of an abdominal painful mass suggests intracystic bleeding. Intraperitoneal bleeding causes abdominal distension and hemorrhagic shock.

 Management

Intracystic bleeding is typical especially to cephalic locations of pancreatic pseudocyst. Bleeding may exteriorize in the digestive tract or intra- / retro-peritoneal space. The source can be arterial, venous or a pseudoaneurysm in the pseudocyst wall. The clinical picture is that of gastrointestinal or intraperitoneal bleeding accompanied by the development of a painful pulsatile epigastric tumour mass.

 The diagnosis and therapeutic option of choice in the centres with logistical facilities in this respect consists of an angiographic embolization of the bleeding source (celiac trunk or superior mesenteric artery). Diagnosis supplementation is made by using abdominal ultrasound scan, CT and upper GI endoscopy [**6,7**].

 Despite the multitude of diagnosis methods, the situation the diagnosis or bleeding cause remains obscure or attributed to another disease is not rare, laparotomy being the only way to make the accurate diagnosis. 

 The therapeutic approach aims at controlling the bleeding source, which can be achieved by both radiology (embolization) and surgery, in hemodynamically unstable patients.

 Studies in the specialty literature show a higher mortality in the case of acute pancreatic pathologies (60% vs. 22% in the case of chronic pathologies), in the case of cephalic location of lesions (43% vs. 15%), in the case of emergency surgeries (33% vs. 12%), in case of re-bleeding [**4,14,,15**].

## Conclusion 

Evolution to complications of pancreatic pseudocyst - bleeding, rupture, and abscess - is of 14% with a mortality of 60% [**10,12**]. Following the evolution of its size and endoscopic or surgical drainage of pseudocysts, if there are signs of progression there is a compulsory condition in preventing the appearance of the three types of complications whose appearance has a difficult treatment solution and a highly associated mortality.
